# Prevalence of Dental Abnormalities, Soft Tissue Pathologies and Occlusion Disorders in Patients with High BMI: A Cross-sectional Study

**DOI:** 10.3290/j.ohpd.b5656148

**Published:** 2024-08-06

**Authors:** Amal Adnan Ashour, Ali Abdullah Alqarni

**Affiliations:** a Associate Professor, Department of Oral and Maxillofacial Surgery and Diagnostic Sciences Faculty of Dentistry, Taif University, Taif, Saudi Arabia. Study design and methodology, interviewed/examined participants, wrote the manuscript.; b Assistant Professor, Department of Oral and Maxillofacial Surgery and Diagnostic Sciences Faculty of Dentistry, Taif University, Taif, Saudi Arabia. Study design and methodology, wrote the manuscript, performed statistical analysis.

**Keywords:** body mass index, dental abnormalities, obesity, oral mucosal lesions, Saudi female

## Abstract

**Purpose:**

The relationship between body mass index (BMI) and oral disorders remains unclear. This study examined the prevalence and types of dental abnormalities and oral mucosal lesions among female students with obesity attending a Taif University sports centre.

**Materials and Methods:**

This non-interventional cross-sectional study enrolled female students with high BMI from a university sports facility using a convivence sampling method. The participants were divided into three BMI groups. Data were collected using an interview and by clinical oral examination. Prevalence and oral disorder types and possible mechanisms linking BMI and dental development were evaluated.

**Results:**

Ultimately, 86 female students with obesity were analysed. The mean BMI was 42.8 kg/m^2^, indicating high obesity levels. A weak although statistically significant correlation was observed between age and BMI (r=0.27), indicating that older students had higher BMI. A statistically significant association was observed between BMI and dental abnormalities (p<0.05). The dental abnormality prevalence increased with BMI, ranging from 37.5% to 40.7% in the ≤40 and >45 kg/m^2^ groups, respectively. Most participants (66.3%) had oral mucosal lesions, with the highest prevalence among participants in the 40–45 kg/m^2^ group (71.4%).

**Conclusion:**

A statistically significant relationship was observed between BMI and dental abnormalities; obesity may negatively affect oral health.

The body mass index (BMI) is a measure of body fat based on height and weight. Obesity class I corresponds to a BMI ≥30–34.99 kg/m^2^, class II to a BMI ranging from 35–39.99 kg/m^2^, and class III (morbid obesity) to a BMI ≥40 kg/m^2^.^33^ BMI is widely used as an indicator of health status and chronic disease risk.^16,29^ However, BMI may also affect oral health and dental development.^17,34^ BMI is associated with dental caries, periodontal disease, tooth loss, and oral cancer.^2,14^ However, the relationship between BMI and dental abnormalities and oral mucosal lesions has not been well explored. People with obesity and overweight are at high risk for developing many diseases and health conditions, including hypertension, dyslipidaemia (i.e., high low-density lipoprotein cholesterol, low high-density lipoprotein cholesterol, or high triglyceride levels), type 2 diabetes mellitus, coronary heart disease, stroke, gall bladder disease, osteoarthritis, sleep apnoea and respiratory problems, and some cancers.^30^ In addition, obesity has negative effects on oral health as well as psychological, social, and economic aspects. Obesity is a systemic illness that increases a person’s risk for numerous co-morbidities and problems that have a negative impact on both general and oral health.^30^ Periodontal diseases, caries, xerostomia, and mucosal changes are examples of oral disorders affected by obesity and overweight.^27^

Dental abnormalities are deviations from the normal shape, size, number, or arrangement of teeth and can affect the function, appearance, and oral health of individuals.^1,15^ Common dental abnormalities include missing teeth, supernumerary teeth, peg-shaped teeth, and malocclusion.^20^ Although the aetiologies of dental abnormalities are not fully understood, they may involve genetic, environmental, and nutritional factors.^1,10^ Amelogenesis imperfecta is a group of hereditary disorders that affects enamel formation and mineralisation, resulting in hypo-plastic or hypo-mineralised enamel.^12^ Both types of amelogenesis imperfecta may increase susceptibility to caries and tooth wear.^12,15^ In addition, it was found that obesity was associated with malocclusion in female subjects.^28^ There is no evidence in the literature about the causal relation of dental abnormalities with obesity. However, dental abnormalities may possibly lead to obesity because of their negative impact of on mental health.

Oral mucosal lesions are a group of disorders that affect the lining of the mouth and may present with various signs and symptoms such as pain, bleeding, ulceration, swelling, or mucosal discolouration, gingivitis and aphthosis, buccal mucosal chewing, candidiasis, and hairy tongue.^8,11^ Oral mucosal lesions can have different aetiologies, such as infectious, inflammatory, immune-mediated, neoplastic, traumatic, or congenital causes.^18^ Some oral mucosal lesions are benign and self-limiting, whereas others may be potentially malignant or indicative of systemic diseases. Radwan-Oczko et al^26^ stated that the highest prevalence of oral mucosal lesions was associated with female gender in the southwestern part of Poland. Evidence exists that obesity is a risk factor for oral mucosal lesions,^19^ and that female hormones could be related to their development.^24^

According to a study by Taghat et al,^33^ young obese women with different BMIs showed poor oral health and high rates of caries in relation to their BMI. Saudi Arabia has a high prevalence of obesity, ranking 29th in the world and third among Arab countries. Moreover, approximately 36% of university students in Saudi Arabia are obese.^23^

In Saudi Arabia, the prevalence of high BMI among females has been a growing concern.^6^ Recent studies have indicated that the prevalence of overweight and obesity continues to rise, with estimates suggesting that more than 60% of adults, including women, are affected by obesity or overweight conditions.^6^ This trend is alarming and underscores the need for effective public health interventions and awareness programs tailored to the female population in the region.^32^

The purpose of this study is to examine the link between body mass index and oral health in Saudi women, with a particular focus on dental anomalies and oral mucosal diseases. We will investigate the prevalence of high BMI in this group, as well as any possible links connecting BMI and dental anomalies, soft tissue diseases, and occlusion alterations. Furthermore, we intend to compare our findings to current research from other locations to detect any distinctive trends or inequalities in Saudi Arabia.

## MATERIALS AND METHODS

This study used a non-interventional cross-sectional design to examine the relationship between high BMI and dental abnormalities, soft tissue pathologies and occlusion disorders in female students at Taif University sports centre. This study was approved by the institutional review board of Taif University (ethical clearance number: 44–131) and followed the principles of the Declaration of Helsinki. Written informed consent was obtained from all participants.

The target population was female students with obesity enrolled at Taif University during the 2022–2023 academic year. The inclusion criteria were female sex, enrolled at TAIF University sports centre and a BMI of ≥29 kg/m^2^ (which is considered overweight/obese according to the World Health Organization standards). The exclusion criterion was BMI <29 kg/m^2^. A convenience sampling method was used to recruit participants for this study. Convenience sampling is a non-probability sampling technique that involves selecting participants who are easily accessible and willing to participate. The participants were recruited through posters, flyers, and social media announcements that invited female students with BMIs of ≥29 kg/m^2^ to participate in the study.

The data collection process comprised two parts: an interview and a clinical oral examination. The interview was conducted by the examiners. The interview included questions about the participants’ demographic characteristics (age, sex, and educational level), oral health impacts on the quality of life (oral symptoms, functional limitations, emotional well-being, and social well-being), and dietary habits. The interview was adapted from the Oral Health Impact Profile, a validated instrument that measures the impact of oral health on quality of life.^31^ Clinical oral examinations were performed by a trained and calibrated dentist at the dental clinic of the sports facility. Diagnosis of the participants’ oral mucosal lesions and dental abnormalities was established according to World Health Organization guidelines.^36^ The examination was conducted using standard dental instruments, such as mouth mirrors, probes, explorers, and periodontal probes.

Descriptive and inferential data analyses were performed. Descriptive statistics were used to describe the participants’ characteristics and their responses to the questionnaire and clinical examination. Descriptive statistics included frequencies and percentages for categorical variables and means and standard deviations or medians and interquartile ranges for continuous variables, depending on the data distribution. Inferential statistics were used to test the hypotheses and answer the research questions. Inferential statistics included contingency table arrays and the χ^2^ statistic for examining categorical variables, and linear or logistic regression analyses for identifying potential predictors of severe periodontitis. The associations between predictors and outcomes were presented as beta (β) and 95% confidence intervals (CIs) for linear regression analyses, and as odds ratios (OR) and CIs for logistic regression analyses. All statistical significance testing was two-tailed with a p-value of 0.05. Data were analysed using STATA Statistical Software (release 15, StatCorp; College Station, TX, USA). The regression analyses results are presented before and after adjusting for potential confounders.

## RESULTS

### Sample Characteristics

**Fig 1:**
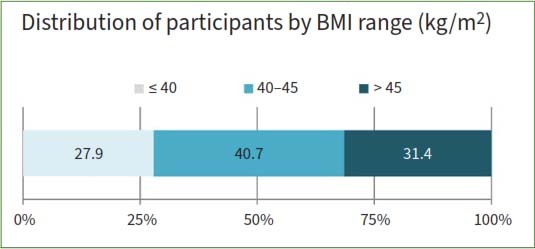
Distribution of participants by body mass index (BMI) range (kg/m^2^).

The sample consisted of 86 female students with obesity; the average age was 21 ± 2.4 years (range 18–27 years.) As shown in Table 1, the participants were divided into three BMI groups (Fig 1). The majority of participants belonged to the 40–45 kg/m^2^ group. The sample size was achieved based on a review of the pertinent literature. A formula based on that of Coto et al^13^ was used to calculate required sample size

**Table 1:** Participant characteristics according to body mass index range

Parameter		BMI range			
		29–40 kg/m^2^	40–45 kg/m^2^	>45 kg/m^2^	Total
Number (n)		24	35	27	86
Percentage (%)		27.9	40.7	31.4	100.0
Age (in years)	Mean	20.3	20.9	21.9	21.0
	SD	1.9	2.5	2.6	2.4
Weight	Mean	94.4	100.5	111.2	102.2
	SD	6.2	9.1	13.1	11.9
Height	Mean	1.58	1.54	1.52	1.54
	SD	0.05	0.07	0.08	0.07
Smoking	No	24 (100%)	32 (91.4%)	24 (88.9%)	80 (93%)
	Yes	0	3 (8.6%)	3 (11.1%)	6 (7%)

BMI: body mass index; SD: standard deviation.

As shown in Table 1, the participants had a mean BMI of 42.8 kg/m^2^, indicating a high level of obesity. The data also revealed that 27.9% of the participants had a BMI of ≤40 kg/m^2^, suggesting that they were overweight or obese but not severely obese. Moreover, 40.7% of participants had a BMI of 40–45 kg/m^2^, indicating severe obesity, and 31.4% had a BMI >45 kg/m^2^, indicating very severe obesity. These results indicate that the sample had a high prevalence of obesity, which may have implications for health and well-being.

**Fig 2:**
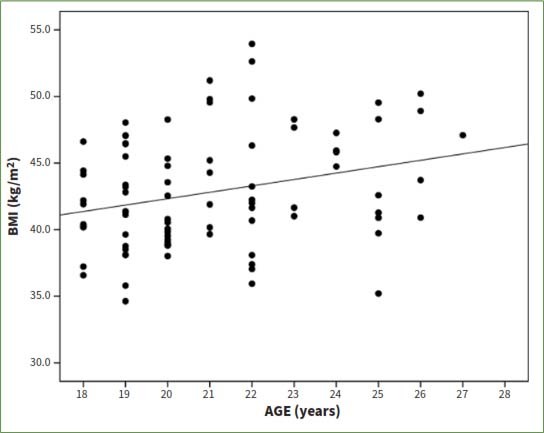
Positive correlation between body mass index (BMI) and age (Spearman r = 0.27; p = 0.012*).

The mean weight of the participants was 102.2 ± 11.9 kg. However, the mean value of the height in the sample was 1.54 ± 0.07 m. A weak albeit statistically significant correlation was observed between age and BMI (r = 0.27): the older the student, the higher the BMI (Fig 2).

### Dental Abnormalities

Table 2 shows the association between the BMI range and dental abnormalities. The analysis revealed a statistically significant difference in the prevalence of dental abnormalities between the BMI groups (p<0.05). The proportion of participants with dental abnormalities increased with higher BMI, ranging from 37.5% in the ≤40 kg/m^2^ group to 40.7% in the >45 kg/m^2^ group.

**Table 2:** Variation in the occurrence of different dental abnormalities among the participants by BMI range group

Dental abnormalities		BMI range								p-value
		Total		29–40 kg/m^2^		40–45 kg/m^2^		>45 kg/m^2^		
		N	%	n	%	n	%	n	%	
Hypo-calcified amelogenesis imperfecta	No	80	93.0	21	87.5	32	91.4	27	100.0	0.49
	Yes	6	7.0	3	12.5	3	8.6	0	0	
Amelogenesis imperfecta hypo-maturation type	No	80	93.0	22	91.7	32	91.4	26	96.3	0.78
	Yes	6	7.0	2	8.3	3	8.6	1	3.7	
Dentinogenesis imperfecta	No	83	96.5	23	95.8	35	100.0	25	92.6	0.68
	Yes	3	3.5	1	4.2	0	0	2	7.4	
Dental abnormalities	Total	86	100.0	24	100.0	35	100.0	27	100.0	0.73
	No	55	64.0	15	62.5	24	68.6	16	59.3	
	Yes	31	36.0	9	37.5	11	31.4	11	40.7	

BMI: body mass index.

The results indicated that the prevalence of dental abnormalities increased with higher BMI, from 37.5% in the ≤40 kg/m^2^ group to 40.7% in the >45 kg/m^2^ group. The most frequent dental abnormalities observed in the participants were hypo-maturation amelogenesis imperfecta (7%), and hypo-calcified amelogenesis imperfecta (7%). These anomalies may have different aetiologies and mechanisms, although they may also share some common risk factors for obesity. For instance, Angle’s Class II malocclusion may be influenced by genetic, environmental, and behavioural factors, such as mouth breathing, thumb sucking, and soft diet, which may also affect the development of obesity.^1^

### Occlusion Disorders

Table 3 shows that the prevalence of general occlusal abnormalities was 36.0% overall, with a slightly higher incidence in the >45 kg/m^2^ group (40.7%). Angle’s Class II malocclusion was present in 8.1% of the total population, with an increased prevalence of 14.8% in the >45 kg/m^2^ BMI range (Table 4). Crossbite was observed in 4.7% of the total sample, with the highest occurrence in the >45 kg/m^2^ group at 11.1%. Angle’s Class III malocclusion was noted in 5.8% of participants, with the lowest prevalence in the >45 kg/m^2^ group (3.7%).

**Table 3:** Variation in the occurrence of different occlusion abnormalities among the participants by BMI range group

Occlusion abnormalities		BMI range								p-value
		Total		29–40 kg/m^2^		40–45 kg/m^2^		>45 kg/m^2^		
		N	%	n	%	n	%	n	%	
Angle’s Class II	Total	86	100.0	24	100.0	35	100.0	27	100.0	0.39
	No	79	91.9	24	100.0	32	91.4	23	85.2	
	Yes	7	8.1	0	0	3	8.6	4	14.8	
Crossbite	Total	86	100.0	24	100.0	35	100.0	27	100.0	0.34
	No	82	95.3	23	95.8	35	100.0	24	88.9	
	Yes	4	4.7	1	4.2	0	0	3	11.1	
Angle’s Class III	Total	86	100.0	24	100.0	35	100.0	27	100.0	0.77
	No	81	94.2	22	91.7	33	94.3	26	96.3	
	Yes	5	5.8	2	8.3	2	5.7	1	3.7	
	No	55	64.0	15	62.5	24	68.6	16	59.3	
	Yes	31	36.0	9	37.5	11	31.4	11	40.7	

BMI: body mass index.

**Table 4:** Variation in the occurrence of different oral mucosal lesions among the participants by BMI range group

Oral mucosal lesion		BMI range								p-value
		Total		≤40 kg/m^2^		40–45 kg/m^2^		>45 kg/m^2^		
		N	%	N	%	N	%	N	%	
Gingivitis	Total	86	100.0	24	100.0	35	100.0	27	100.0	0.45
	No	64	74.4	19	79.2	25	71.4	20	74.1	
	Yes	22	25.6	5	20.8	10	28.6	7	25.9	
Aphthosis	Total	86	100.0	24	100.0	35	100.0	27	100.0	0.82
	No	69	80.2	20	83.3	27	77.1	22	81.5	
	Yes	17	19.8	4	16.7	8	22.9	5	18.5	
Buccal mucosa chewing	Total	86	100.0	24	100.0	35	100.0	27	100.0	0.74
	No	79	91.9	22	91.7	33	94.3	24	88.9	
	Yes	7	8.1	2	8.3	2	5.7	3	11.1	
Mucosal discolouration	Total	86	100.0	24	100.0	35	100.0	27	100.0	0.29
	No	74	86.0	21	87.5	32	91.4	21	77.8	
	Yes	12	14.0	3	12.5	3	8.6	6	22.2	
Candidiasis	Total	86	100.0	24	100.0	35	100.0	27	100.0	0.47
	No	78	90.7	21	87.5	31	88.6	26	96.3	
	Yes	8	9.3	3	12.5	4	11.4	1	3.7	
Hairy tongue	Total	86	100.0	24	100.0	35	100.0	27	100.0	0.96
	No	84	97.7	24	100.0	34	97.1	26	96.3	
	Yes	2	2.3	0	.0	1	2.9	1	3.7	
Oral mucosal lesion	Total	86	100.0	24	100.0	35	100.0	27	100.0	0.70
	No	29	33.7	9	37.5	10	28.6	10	37.0	
	Yes	57	66.3	15	62.5	25	71.4	17	63.0	

BMI: body mass index.

### Oral Mucosal Lesions

Table 3 shows the association between the BMI range and oral mucosal lesions. The analysis revealed that every 1-kg/m^2^ increase in BMI increased the risk of lesions by 9% (odds ratio=1.09; p=0.195), but not statistically significantly (p=0.195).

The majority of the participants (66.3%) had oral mucosal lesions, with the highest prevalence among those with BMIs ranging from 40–45 kg/m^2^ (71.4%). No statistically significant difference was observed in the occurrence of oral mucosal lesions among the BMI groups.

#### Gingivitis

Approximately one-quarter of the participants (25.6%) had gingivitis, with no statistically significant differences among BMI groups. The lowest prevalence of gingivitis was among those with a BMI ≤40 kg/m^2^ (20.8%).

#### Aphthosis

Less than one-fifth of the participants (20%) had aphthosis, with the highest prevalence among those with a BMI ranging from 40–45 kg/m^2^ (23%). No statistically significant differences in the occurrence of aphthosis were observed among BMI groups.

#### Other oral conditions

The prevalence of other oral conditions, such as buccal mucosal chewing, discolouration, candidiasis, and hairy tongue, was low (<15%) among the participants, with no statistically statistically significant differences among BMI groups. The highest prevalence of discolouration was observed among those with a BMI >45 kg/m^2^ (22.2%).

## DISCUSSION

This study evaluated the association between obesity and oral health changes in female students with obesity visiting a sports facility at Taif University of Saudi Arabia. The results showed that the sample had a high prevalence of obesity, which may have implications for health and well-being. According to the World Health Organization, obesity is defined as a BMI of ≥30 kg/m^2^, and is associated with an increased risk of various chronic diseases, such as cardiovascular disease, diabetes, and some cancers.^5^ Moreover, obesity can affect the quality of life and psychological well-being of individuals as they may experience stigma, discrimination, and low self-esteem.^29^

The mean BMI of the sample was 42.8 kg/m^2^, indicating a high level of obesity among the participants. This is consistent with previous studies reporting a high prevalence of obesity among Saudi women.^4,9,21^ The data also revealed that 27.9% of the participants had a BMI of ≤40 kg/m^2^, suggesting that they were overweight or obese but not severely obese. However, 40.7% of participants had a BMI of 40–45 kg/m^2^, indicating severe obesity, and 31.4% had a BMI >45 kg/m^2^, indicating very severe obesity. These results indicate that most participants had extreme forms of obesity, which may have more adverse effects on their health and oral status than moderate obesity.

Our results also showed that the most common oral mucosal lesions in this study were gingivitis and aphthosis, followed by other conditions such as buccal mucosal chewing, mucosal discolouration, candidiasis, and hairy tongue. These findings are similar to those reported by other studies.^18,35^ The prevalence of gingivitis and aphthosis in this study was higher than that of other lesions, which could be attributed to poor oral hygiene, smoking, stress, and nutritional deficiencies among the participants.

Since this was a cross-sectional study, establishing causal relationships was difficult, and the observed associations might be due to other unexplored factors. Recall bias related to genetic and environmental causes cannot be ruled out. The results showed a statistically significant association between BMI and dental abnormalities. These findings are consistent with those of some previous studies although inconsistent with others, suggesting that the relationship between obesity and oral health is complex and multi-factorial. No previous studies have investigated the relationship between amelogenesis imperfecta and obesity in Saudi Arabia. The association between amelogenesis imperfecta and obesity may be explained by the involvement of genes that regulate enamel formation and energy metabolism.^12,15^ These findings show that obesity may have a negative impact on oral health, warranting further investigation into the causal mechanisms and preventive measures.

Similarly, the impact of BMI on soft tissue pathologies and occlusion changes has been the subject of research. There is evidence to suggest that a higher BMI may be associated with certain oral health indicators, such as periodontitis, tooth loss, and caries, due to common risk factors (e.g, dietary habits, genetics, and lifestyle choices).^2,3^ However, the data specific to Saudi Arabia, and particularly the region of Taif University where the study was conducted, remains limited. This gap in research highlights the need for localised studies to better understand the implications of BMI on dental and soft tissue health within the Saudi population.

The extant literature lacks empirical investigations into the correlation between dental malocclusion and elevated BMI. The findings from the cross-sectional analysis suggest that dental occlusion factors exhibit no statistically significant relationship with the intensity of obstructive sleep apnea (OSA) in non-obese adult patients within the Saudi demographic.^7^ The prevalence of buccal or lateral cross-bite, attributable to maxillary skeletal constriction, was noted in approximately 30%–50% of the OSA cohort, independent of the measured severity.^7^ Notwithstanding, this study revealed a prevalent association of OSA with anterior cross-bite. While a discernible variance in the distribution of cross-bite across patients with varying degrees of OSA severity was observed, the results were not statistically significant.^7^ Vigilant monitoring of BMI in children is imperative for the evaluation of dental health and the formulation of treatment regimens for individuals aged 6 to 14.^14,25,29^ Understanding the interplay between BMI and the chronology of permanent tooth eruption equips dental practitioners with the ability to discern patterns indicative of either precocious or belated development.^22^ This knowledge is instrumental in devising customised treatment modalities, thereby enhancing the precision of oral health interventions and outcomes for this age bracket.

### Study Limitations

Several limitations should be considered when interpreting our findings. It is important to acknowledge that our study exclusively included female participants, which may limit the generalisability of our findings to broader populations. Additionally, being a single-centre study introduces potential biases related to local demographics, healthcare practices, and institutional factors. Since this was a cross-sectional study, establishing causal relationships was difficult, and the observed associations might be due to other, unexplored factors. Recall bias related to genetic and environmental causes cannot be ruled out. In addition, considering that this investigation constituted a pilot study, the sample size was relatively modest, potentially limiting the statistical power to detect statistically significant findings. Moreover, the small sample size might have resulted in overestimated odds ratios (ORs). To advance the existing literature, further research involving a larger sample of similarly high-risk groups would be advantageous.

## CONCLUSION

This study on female students with obesity at Taif University in Saudi Arabia found a substantial prevalence of severe obesity and a considerable impact on dental health. Higher BMI was linked to more dental abnormalities, such as hypomaturation and hypocalcified amelogenesis imperfecta, in addition to an increased risk of oral mucosal diseases such as gingivitis and aphthosis. Although the cross-sectional methodology restricts causal inferences, the findings highlight the complicated relationship between obesity and oral health, emphasising the importance of integrated health interventions and more study to elucidate these correlations and establish effective preventive measures.
